# Circular plant peptides as templates for GPCR drug discovery?

**DOI:** 10.1186/1471-2210-11-S2-A16

**Published:** 2011-09-05

**Authors:** Johannes Koehbach, Margaret O’Brien, Marion Miazzo, David J Craik, Michael Freissmuth, Christian W Gruber

**Affiliations:** 1 Institute of Pharmacology, Center of Physiology and Pharmacology, Medical University of Vienna, 1090 Vienna, Austria; 2 National Center for Biomedical and Engineering Science, National University of Ireland Galway, Galway, Ireland; 3 Institute for Molecular Biosciences, University of Queensland, St. Lucia, QLD 4072, Australia

## Background

Cyclotides are disulfide-rich miniproteins with the unique structural features of a circular backbone and knotted arrangement of three conserved disulfide bonds. These features make them exceptionally stable and they have applications as plant defense (insecticidal) agents and stable drug frameworks. So far they have been found mainly in two plant families, including in every species of the violet family (*Violaceae*) so far examined, and in a few species of the coffee family (*Rubiaceae*).

## Methods

We optimized the cyclotide screening protocol using MALDI-TOF/TOF and two dimensional nano LC-MS/MS followed by manual and automated peptide sequence assignments. Biological analysis was carried out by collagen uterine contractility assays and pharmacological data were generated by competitive displacement binding and functional GPCR luciferase assays.

## Results

Using the rapid cyclotide discovery workflow, we analyzed >300 flowering plant species and confirmed the presence of cyclotides in several plant families. On the basis of the phylogeny of cyclotide-bearing plants and the analysis of precursor gene sequences, we have refined the current hypothesis of the evolution of circular plant peptides. Based on the traditional usage of the plant *Oldenlandia affinis* as an uterotonic agent, we have tested and identified cyclotides with oxytocin-like activity and identified the oxytocin receptor, as representative member of the GPCR family, as a molecular target of cyclotides.

## Conclusions

Cyclotides are interesting targets for pharmaceutical applications due to their unique structural framework, their bioactivities and sequence diversity. Based on their predicted number of tens of thousands, which potentially makes them one of the largest protein families within the plant kingdom, they constitute an immense combinatorial library of natural peptides, which is accessible for drug discovery.

